# Biochemical profiles of patients with COVID-19 during the first and second waves in Ethiopia

**DOI:** 10.3389/fimmu.2024.1426413

**Published:** 2024-12-10

**Authors:** Habtamu Abebe Getahun, Assefa Legesse, Diliab Desta, Ahmed Johar, Israel Bekele, Kebenesa Angasu, Samuel Hunegnaw, Nebiyou Simegnew, Minale Fekadie

**Affiliations:** ^1^ Department of Epidemiology, Public Health Faculty, Institute of Health, University of Gondar, Gondar, Ethiopia; ^2^ Department of Epidemiology, Public Health Faculty, Institute of Health, Jimma University, Jimma, Ethiopia; ^3^ Department of Anatomy, School of Biomedical Sciences, Jimma University, Jimma, Ethiopia; ^4^ Department of Biomedical Sciences, College of Medicine and Health Sciences, Wollo University, Dessie, Ethiopia; ^5^ School of Nursing, Faculty of Health Sciences, Jimma University, Jimma, Ethiopia; ^6^ Department of Internal Medicine, College of Medicine and Health Sciences, Bahir Dar University, Bahir Dar, Ethiopia; ^7^ Department of Surgery, Faculty of Health Sciences, Institute of Health, Jimma University, Jimma, Ethiopia; ^8^ Department of Biochemistry, School of Biomedical Sciences, Jimma University, Jimma, Ethiopia

**Keywords:** biochemical profiles, waves, COVID-19, patients, Ethiopia

## Abstract

Coronavirus disease 2019 (COVID-19) is a highly infectious disease caused by severe acute respiratory syndrome coronavirus 2. Nasopharyngeal swabs (NP swabs) were used for patients with COVID-19 who demonstrated serious clinical symptoms and disturbances in biochemical parameters. The biochemical profiles of these patients remain ambiguous and differ from wave to wave of COVID-19 infections. Herein, we conducted a multicenter retrospective cohort study with 538 patients with COVID-19 at six COVID-19 treatment centers in Ethiopia. Professional data collectors collected the data. Descriptive statistics were used to summarize the data, and independent t-tests and chi-square tests were used to assess the relationships between the continuous and categorical variables across waves, respectively. In total, 240 and 298 patients were included from the first and second waves, respectively. Men and individuals aged 53–69 years were more likely to be infected in each wave. The mean alkaline phosphatase (p < 0.001) and sodium levels (p = 0.035) significantly differed between patients across the two waves of COVID-19; the significant difference in the alkaline phosphatase levels of patients between the two waves was −45.425. All the symptoms of COVID-19 were significantly (p < 0.05) associated with the waves of the pandemic. Patients in both waves had no chronic disease comorbidities. This study showed that the mean alkaline phosphatase and sodium levels differed significantly across the first two waves of the pandemic at six COVID-19 treatment centers in Ethiopia while all clinical symptoms of COVID-19 were associated with the first two waves of the pandemic.

## 1 Introduction

Severe acute respiratory syndrome coronavirus 2 (SARS-CoV-2), which causes coronavirus disease 2019 (COVID-19), is a member of a large family of enveloped-positive single-stranded RNA (ribonucleic acid) viruses of medical importance (1). The virus particle exhibits a characteristic “corona” (crown) of spike proteins around the lipid envelope, which is the basis of these viruses being referred to as coronaviruses (2). Coronaviruses are a large family of viruses that cause ailments ranging from asymptomatic illness to illnesses with high morbidity and mortality, including Middle East respiratory syndrome and severe acute respiratory syndrome (3).

The 2019 novel coronavirus produced a new illness that was first recognized in human beings in Wuhan, China in December 2019 and swiftly spread to become a pandemic (4). The World Health Organization (WHO) declared a public health emergency on 30 January 2020 (5). On 11 March 2020, the WHO declared the disease a pandemic (6). According to a worldwide report on 18 November 2021, the virus had spread to >222 countries and territories. Altogether, 255,772,102 laboratory-confirmed infections and 5,139,858 confirmed deaths have been reported worldwide (5). In 2024, COVID-19 remains a monitored global health issue, though prevalence rates and variant distributions vary by region. WHO reports show that in recent months, global COVID-19 cases have decreased in most areas, with slight increases in others, particularly in countries in the Western Pacific and some parts of Africa (7).

The disease is highly infectious, and patients with COVID-19 demonstrate serious clinical symptoms, including increased body temperature, dry cough, diarrhea, headaches, myalgia, fever, fatigue, dyspnea, vomiting, anorexia, and multiple organ dysfunction (4, 8). The clinical manifestations of COVID-19 vary from asymptomatic to acute respiratory distress, depending on the viral route of entry, viral load, host immunity, age, and comorbidities such as cardiovascular disease, diabetes, chronic respiratory disease, and cancer (8). The severe stage of the disease can be characterized by acute respiratory distress syndrome, treatment-resistant septic shock, metabolic acidosis, and bleeding and coagulation dysfunction (9, 10).

The current reporting on laboratory-confirmed COVID-19 cases reports changes in the biochemical parameters of patients (i.e., levels of ferritin, CRP, IL-6, LDH, AST, ALT, PT, D-dimer, procalcitonin, PLT dimer, and APTT) (11). Therefore, these biochemical parameters can be used as predictive markers. Many studies have proven that comorbidities, age, and abnormalities together with numerous scientific biomarkers may be essential for understanding illness severity (12). Although the clinical characteristics of COVID-19 have been widely reported, a summary of the changes in the common biochemical variables observed in patients infected during different waves of COVID-19 remains poorly investigated. Published laboratory findings of COVID-19 infections have been collected from hospital-admitted patients at single time points, which limits our understanding of the dynamic biochemical changes during the course of the disease and how clinical symptoms and disease severity differ across waves (13). Thus, the consistency and differences in the biochemical parameters of patients with COVID-19 across waves remain largely unknown. Therefore, this study assessed the biochemical profiles of patients with COVID-19 during the first and second waves of the pandemic in Ethiopia.

## 2 Methods

### 2.1 Study design, setting and population

A multicenter retrospective cohort study was conducted at six COVID-19 treatment centers in Ethiopia [Jimma University Medical Center (JUMC), St. Peter’s Specialized Hospital, EKA Kotebe General Hospital, Bethzatha General Hospital, Hallelujah General Hospital, and Bahir Dar University Tibebe Ghion Specialized Hospital] to assess the biochemical profiles of patients with COVID-19. A total of 538 study participants (COVID-19 PCR- and RDT-positive) were recruited from the selected COVID-19 treatment centers in Ethiopia and the WHO Structured Laboratory Testing and Reporting Checklist was used.

### 2.2 Data collection tool and procedure

Data were collected using a structured checklist. The checklist was adapted from the WHO tool for data extraction and developed after an extensive review of the literature and similar study tools (14, 15). Professional and experienced data collectors and supervisors were selected and trained by the principal and co-investigator for data collection. They practiced using the data collection tool with the principal investigator for 2 days before starting official data extraction. Data were then gathered from both electronic and paper medical records of suspected and confirmed COVID-19 cases. Information collected included demographic details (age, sex, educational status, ethnicity, nationality, and marital status), medical history (comorbidities), clinical signs and symptoms, physical examination results, COVID-19 status, and hospital stay details. Additionally, laboratory data, patient demographic information, medical histories, and biochemical profiles [direct bilirubin, urea, blood urea nitrogen, total bilirubin, blood glucose, cholesterol, low-density lipoprotein (LDL), Serum Glutamate Pyruvate Transaminase / Alanine Aminotransferase (measured in units per liter) (SGPT/ALT U/L), Serum Glutamate Oxaloacetate Transaminase / Aspartate Aminotransferase (measured in units per liter) (SGOT/AST U/L), gamma-glutamic, transpeptidase, alkaline phosphatase, sodium (NA^+^), potassium (K^+^), chloride (Cl^-^), phosphorus, magnesium, triglycerides, high-density lipoprotein (HDL), and calcium (Ca^+^)] were extracted from these records.

#### 2.2.1 Data quality control and statistical analysis

The selected data collectors were trained to collect data from medical records. Facilitators and supervisors were assigned to control and guide data collection, thereby increasing consistency in data collection. Collected data were checked for completeness and consistency before entry and double data entry into EpiData 4.6. Double data entry is a data management method used to improve data accuracy and reduce entry errors. In this process, two independent data entry operators enter the same dataset separately, creating two versions. These entries are then compared, and any discrepancies between the two are reviewed and resolved. After checking the data, it was exported to SPSS version 25. Descriptive statistics were used to determine frequencies with percentages and means. Graphs, charts, and tables were used to summarize the data. Independent t-tests were used to compare the mean biochemical and hematological profiles of the patients with COVID-19, and chi-square tests were conducted to identify associations between categorical variables across the waves.

### 2.3 Ethical considerations

Ethical clearance and approval for this research were obtained from the Institutional Review Board of Jimma University (Ref. No. JUIRB43/22). The directors of the selected COVID-19 treatment centers were contacted via a formal letter by the director’s office of the Institute of Health, Research, and Innovation at Jimma University. Supporting letters were secured from each treatment center. All participants were informed of the objectives of the study, and written informed consent was obtained before starting data collection.

## 3 Results

### 3.1 Sociodemographic characteristics of participants

A total of 538 patients with COVID-19 were included in the analysis: 240 (44.6%) from the first wave and 298 (55.4%) from the second wave. The mean age in the first wave was 56 ± 2 years, and ranged from 1 to 97; the mean age in the second wave was 56 ± 1 years, and ranged from 16 to 95. An unequal sex distribution was observed in both sets of patients; furthermore, the education and marital status significantly differed between both waves (p < 0.05). Increased age was evident during the first wave but not during the second wave. During the first wave of COVID-19, of the 240 patients, 93 (38.75%) had hypertension, 83 (34.58%) had diabetes mellitus, 44 (18.33%) had chronic liver disease, and 31 (12.92%) had a history of ischemic heart disease. During the second COVID-19 wave, of the 298 patients, 116 (38.93%) had hypertension, 98 (32.89%) had diabetes mellitus, and 37 (12.41%) had a medical history of ischemic heart disease (**Table 1**).

**Table 1:** The distribution and association socio-demographic characteristics and medical history of COVID-19 patients across Waves in Ethiopia.

Variables		Waves	
		Wave 1 (N=240) N (%)	Wave 2 (N=298) N (%)
Gender	Male Female	162(67.5%) 78(32.5%)	183(61.4%) 115(38.6%)
Age	<18 19–35 36–52 53–69 ≥70 missing	5(2.08%) 19(7.91%) 67(27.91%) 86(35.83%) 60(25.0%) 3(1.25%)	1(0.34%) 32(10.74%) 84(28.19%) 109(36.58%) 71(23.83%) 1(0.34%)
Educational status	Illiterate Literate Missing	26(10.83%) 29(12.083%) 175(72.92%)	43(14.43%) 87(29.2%) 82(27.52%)
Marital status	Married Unmarried Missing	67(27.92%) 5(6.9%) 158(65.83%)	125(41.95%) 5(3.8%) 82(27.52%)
Chronic lung disease	yes no unknown	7(2.91%) 221(92.08%) 12(5%)	9(3.02%) 272(91.28%) 17(5.70%)
WHO clinical stage	Moderate Severe critical	72(30.10%) 132(55.20%) 35(14.60%)	81(27.20%) 173(58.00%) 44(14.80%)
AIDS/HIV-positive	yes no unknown	10(4.17%) 215(89.6%0 15(6.25%)	7(2.35%) 276(92.62%) 15(5.03%)
Hematologic malignancy	yes no unknown	4(1.70%) 226(94.20%) 10(4.2%)	9(3.02%) 270(90.60%) 19(6.4%)
Chronic liver disease	yes no unknown	4(1.7%) 227(94.6%) 9(3.8%)	3 (1.0%) 276(92.6%) 19(6.4%)
Deep venous thrombosis	yes no unknown	5(2.08%) 231(96.25%) 4(1.67%)	4(1.34%) 275(92.28%) 19(6.34%)
Hypertension	yes no unknown	93(38.75%) 145(60.42%) 2(0.83%)	116(38.93%) 176(59.06%) 6(2.01%)
Ischemic heart disease	yes no unknown	31(12.92%) 198(82.5%) 11(4.6%)	37(12.41%) 244(81.88%) 17(5.05%)
Diabetes Mellitus	yes no unknown	83(34.58%) 156(65%) 1(0.42%)	98(32.89%) 192(64.43%) 8(2.69%)
Asthma	yes no unknown	15(6.25%) 221(92.08%) 4(1.67%)	22(7.38%) 261(87.6%) 15(5.03%)
Viral hepatitis	yes no unknown	1(0.42%) 227(94.6%) 12(5.0%)	3(1.0%) 277(92.95%) 18(6.04%)
Neurological disorder	yes no Unknown	10(4.17%) 224(93.33%) 6(2.5%)	6(2.01%) 266(89.26%) 26(8.72%)^1^

^1^Milligram/.

### 3.2 The clinical stage of patients with COVID-19 across waves

**Figure 1:**
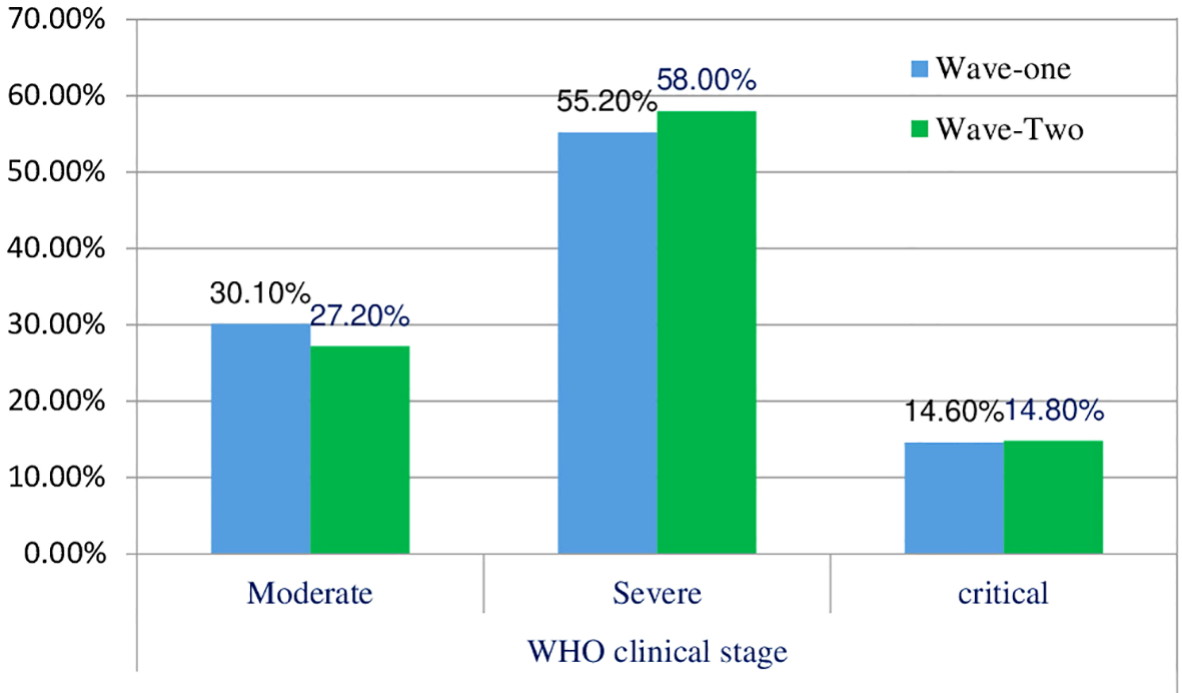
Clinical stages of COVID-19 diseases across the two waves.

**Figure 2:**
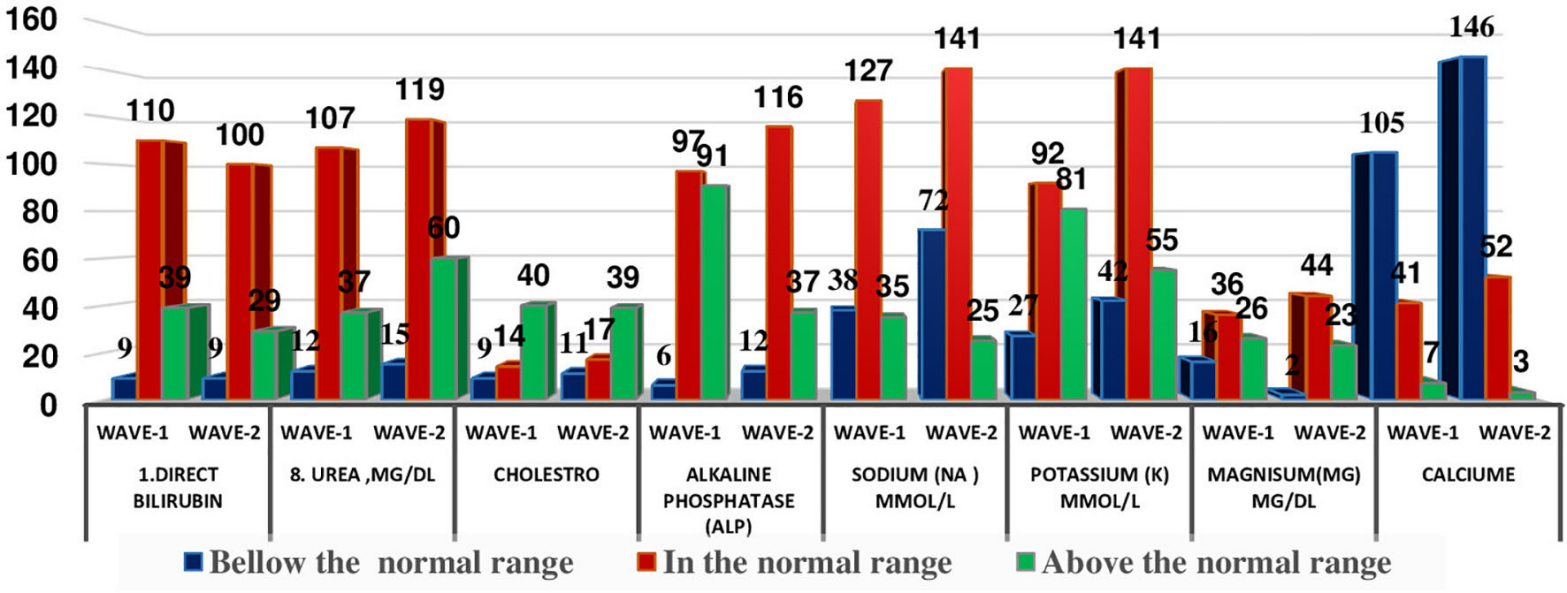
Distribution of some selected biochemical parameters across the two COVID-19 waves in Ethopia.

The distribution of patients in different WHO clinical stages (16) and across the first and second waves of COVID-19 in Ethiopia indicated that of 240 patients, 72 (30.10%) were in the moderate clinical stage, 132 (55.2%) were in the severe clinical stage, and 35 (14.6%) were in the critical stage during the first wave. Of the 298 patients in the second wave, 82 (27.2%) were in the moderate clinical stage, 173 (58.00%) were in the severe clinical stage, and the remaining 44 (4.8%) were in the critical stage (**Figure 1**). The biochemical parameters of the patients with COVID-19 in the selected COVID-19 treatment centers were analyzed. The biochemical parameter recorded in the highest number of patients was Na^+^ in the second wave, whereas the biochemical parameter recorded in the lowest number of patients was the Cl^-^ level in the first wave. The Ca^+^ levels in the second wave were below the normal range. The K^+^ and Na^+^ levels in most patients during the second wave were within the normal range. In the first and second waves of COVID-19, 110 (69.6%) and 100 (72.5%) of the patients had direct bilirubin levels that were within the normal range, 9 (5.7%) and 9 (6.5%) were below the normal range, and 39 (24.7%) and 29 (21.0%) were above the normal range, respectively. The overall distributions of the biochemical parameters across the first two waves are shown in **Table 2**, **Figure 2**.

**Table 2:** The overall biochemical profiles of COVID-19 patients across in Ethiopia.

	Normal range		Wave	
			Wave-1(N=240)	Wave- 2(N=298)
			Count (%)	Count (%)
Direct Bilirubin mg/dl	0.1–0.3	Bellow the normal range	9(5.7%)	9(6.5%)
		In the normal range	110(69.6%)	100(72.5%)
		Above the normal range	39(24.7%)	29(21.0%)
Urea, mg/dL	3.4–20.5	Bellow the normal range	12(7.7%)	15(7.7%)
		In the normal range	107(68.6%)	119(61.3%)
		Above the normal range	37(23.7%)	60(30.9%)
Blood Urea Nitrogen (BUN) mg/dL	15 – 45	Bellow the normal range	3(4.2%)	4(6.5%)
		In the normal range	29(40.3%)	34(54.8%)
		Above the normal range	40(55.6%)	24(38.7%)
Total Bilirubin mg/dl	6-24	Bellow the normal range	138(98.6%)	134(97.1%)
		In the normal range	2(1.4%)	3(2.2%)
		Above the normal range	No individuals	1(0.7%)
Blood Glucose mg/dL	50–80	Bellow the normal range	2(1.9%)	1(0.5%)
		In the normal range	2(1.9%)	4(2.0%)
		Above the normal range	101(96.2%)	200(97.6%)
Cholesterol mg/dL	100-129	Bellow the normal range	9(14.3%)	11(16.4%)
		In the normal range	14(22.2%)	17(25.4%)
		Above the normal range	40(63.5%)	39(58.2%)
LDL, mg/dL	< 150	In the normal range	28(73.7%)	51(86.4%)
		Above the normal range	10(26.3%)	8(13.6%)
SGPT/ALT U/L	< 60	In the normal range	1(0.4%)	No individuals
		Above the normal range	225(99.6%)	225(100%)
SGOT/AST U/L	109–245	Bellow the normal range	No individuals	No individuals
		In the normal range	121(54.8%)	118(51.3%)
		Above the normal range	100(45.2%)	112(48.7%)
Gamma-glutamyl transpeptidase (GGT) U/L	5–35	Bellow the normal range	No individuals	1(16.7%)
		In the normal range	11(29.7%)	1(16.7%)
		Above the normal range	26(70.3%)	4(66.7%)
Alkaline phosphatase (ALP) U/L	8-40	Bellow the normal range	6(3.1%)	12(7.3%)
		In the normal range	97(50.0%)	116(70.3%)
		Above the normal range	91(46.9%)	37(22.4%)
Sodium (Na) mmol/L	9-48	Bellow the normal range	38(19.0%)	72(30.3%)
		In the normal range	127(63.5%)	141(59.2%)
		Above the normal range	35(17.5%)	25(10.5%)
Potassium (K) mmol/L	44-147	Bellow the normal range	27(13.5%)	42(17.6%)
		In the normal range	92(46.0%)	141(59.2%)
		Above the normal range	81(40.5%)	55(23.1%)
Chloride (Cl) mmol/L	135-145	Bellow the normal range	22(12.6%)	28(12.1%)
		In the normal range	82(46.9%)	122(52.6%)
		Above the normal range	71(40.6%)	82(35.3%)
Phosphorus(P), mg/dL	3.5-4.5	Bellow the normal range	5(23.8%)	16(19.0%)
		In the normal range	14(66.7%)	59(70.2%)
		Above the normal range	2(9.5%)	9(10.7%)
Magnesium (Mg) mg/dL	95-105	Bellow the normal range	16(20.5%)	2(2.9%)
		In the normal range	36(46.2%)	44(63.8%)
		Above the normal range	26(33.3%)	23(33.3%)
Triglycerides mg/dL	2.5-4.8	Bellow the normal range	32(51.6%)	40(60.6%)
		In the normal range	No individuals	No individuals
		Above the normal range	30(48.4%)	26(39.4%)
HDL mg/dL	1.6-2.2	Bellow the normal range	39(92.9%)	54(91.5%)
		In the normal range	No individuals	No individuals
		Above the normal range	3(7.1%)	5(8.5%)
Calcium mg/dL	8.5-10.2	Bellow the normal range	105(68.6%)	146(72.6%)
		In the normal range	41(26.8%)	52(25.9%)
		Above the normal range	7(4.6%)	3(1.5%)

### 3.3 The clinical symptoms of patients with COVID-19 across waves

The most relevant clinical symptoms recorded during the first wave were dry cough (166; 69.2%), fatigue (153; 63.75%), shortness of breath (148; 61.67%), and fever (116; 48.33%). During the second wave, fatigue (244; 81.88%), dry cough (242; 81.2%), shortness of breath (204; 68.47%), and fever (180; 60.40%) were the most prevalent symptoms of COVID-19 infection in Ethiopia. A chi-square association test was conducted to identify whether the occurrence of COVID-19 symptoms and pandemic waves were associated. This revealed a significant association between the pandemic waves and headache, myalgia, chest pain, dry cough, fatigue, fever, joint pain, enlarged glands, rash, shortness of breath, dark or bloody urine, yellow skin/eyes, stiff neck, difficulty in swallowing, wheezing, diarrhea, and chills/rigors (**Table 3**).

**Table 3:** The prevalence of COVID-19 symptoms among COVID-19 patients across waves in Ethiopia.

Symptoms/signs	Categories	Waves		P-value
		wave 1 (n=240)	wave 2(n=298)	
Headache	Yes	77(32.08%)	115(38.6%)	<0.0001
	No	148(61.67%)	121(40.60%)
	Unknown	15(6.25%)	62(20.8%)
Myalgia	Yes	68(28.33%)	84(28.19%)	<0.0001
	No	145(60.42%)	123(41.27%)
	unknown	27(11.25%)	91(41.28%)
Chest pain	Yes	59(24.6%)	105(35.23%)	<0.0001
	No	172(71.67%)	156(52.35%)
	unknown	9(3.75%)	37(12.2%)
Dry cough	Yes	166(69.2%)	242(81.2%)	0.003
	No	72(30%)	52(17.45%)
	unknown	2(0.83%)	4(1.34%)
Fatigue	Yes	153(63.75%)	244(81.88%)	<0.0001
	No	76(31.67%)	36(12.08%)
	unknown	11(4.6%)	18(6.04%)
Fever	Yes	116(48.33%)	180(60.40%)	<0.0001
	No	120(50%)	100(33.56%)
	unknown	4(1.67%)	18(6.04%)
Joint pain	Yes	28(11.67%)	62(20.80%)	<0.0001
	No	193(80.42%)	191(64.09%)
	unknown	19(7.92%)	45(15.0%)
Enlarged glands	Yes	2(0.83%)	2(0.67%)	<0.0001
	No	216(90.0%)	221(74.16%)
	unknown	22(9.17%)	75(25.17%)
Rash	Yes	5(2.08%)	3(1.0%)	<0.0001
	No	225(93.75%)	228(76.51%)
	unknown	10(4.17%)	67(23.48%)
Shortness of breath	Yes	148(61.67%)	204(68.47%	<0.0001
	No	86(35.83%)	63(21.14%)
	unknown	6(2.5%)	31(10.40%)
Dark or bloody urine	Yes	5(2.08%)	8(2.68%)	<0.0001
	No	216(90.0%)	226(75.84%)
	unknown	19(7.92%)	64(21.48%)
Yellow skin/eyes	Yes	2(0.83%)	1(0.34%)	<0.0001
	No	225(93.75%)	226(75.84%)
	unknown	13(5.42%)	71(23.83%)
Stiff neck	Yes	2(0.83%)	2(0.67%)	<0.0001
	No	224(93.33%)	203(68.12%)
	unknown	14(5.83%)	93(31.21%)
Difficulty swallowing	Yes	4(1.67%)	8(2.68%)	<0.0001
	No	216(90%)	195(65.44%)
	unknown	20(8.33%)	95(31.88%)
Wheezing	Yes	15(6.25%)	15(5.03%)	<0.0001
	No	212(88.33%)	204(68.46%)
	unknown	13(5.42%)	79(26.51%)
Diarrhea	Yes	12(5.0%)	22(7.38%)	<0.0001
	No	218(90.83%)	217(72.82%)
	unknown	10(4.17%)	59(19.8%)
Chills/rigors	Yes	36(15.0%)	35(11.74%)	<0.0001
	No	185(77.08%)	191(64.09%)
	unknown	19(7.92%)	72(24.16%)
Any other significant symptoms?	Yes	36(15%)	81(27.18%)	<0.0001
	No	196(81.67%)	190(3.76%)
	unknown	8(3.33%)	27(9.06%0

### 3.4 Comparison of the biochemical profiles of patients with COVID-19 by independent t-test across waves in Ethiopia

The biochemical profiles of patients who were infected during the first and second waves of COVID-19 in Ethiopia were compared using an independent t-test to evaluate whether the mean biochemical profiles significantly differed across pandemic waves in Ethiopia. Accordingly, the mean alkaline phosphatase and sodium levels were identified as being significantly different between the first two waves of COVID-19. However, the other biochemical parameters did not significantly differ between the first two waves of the pandemic (**Table 4**).

**Table 4:** Comparison of the biochemical profiles of COVID-19 patients by independent t-test across waves in Ethiopia.

Biochemical Profiles	Waves	N	Mean	P-value
Direct Bilirubin mmol/L	wave 1	158	0.3035	0.590
	wave 2	138	0.3448	
Total Bilirubin mmol/L	wave 1	140	0.7223	0.165
	wave 2	138	1.0189	
Urea, mg/dL	wave 1	156	41.3473	0.523
	wave 2	195	44.0871	
Blood Urea Nitrogen (BUN) mg/dL	wave 1	72	35.1808	0.166
	wave 2	62	28.1548	
Random Blood Glucose mg/dL	wave 1	105	188.8103	0.945
	wave 2	205	188.0771	
Cholesterol/dL	wave 1	63	162.7921	0.097
	wave 2	68	138.8969	
Triglyceride, mg/dL	wave 1	62	171.3339	0.511
	wave 2	66	159.8424	
HDL, mg/dL	wave 1	42	36.9238	0.595
	wave 2	59	38.5339	
LDL, mg/dL	wave 1	38	87.5500	0.941
	wave 2	59	88.5983	
SGPT/ALT U/L	wave 1	226	52.4739	0.600
	wave 2	225	48.7258	
SGOT/AST U/L	wave 1	221	48.0043	0.322
	wave 2	230	54.1909	
Gamma-glutamyl transpeptidase (GGT) U/L	wave 1	37	103.4351	0.873
	wave 2	6	95.3333	
Alkaline phosphatase (ALP) U/L	wave 1	194	156.6835	0.000
	wave 2	165	111.2584	
Sodium (Na) mmol/L	wave 1	200	139.2417	0.035
	wave 2	238	137.3588	
Potassium (K) mmol/L	wave 1	200	4.7361	0.260
	wave 2	238	4.3682	
Chloride (Cl) mmol/L	wave 1	175	102.1099	0.925
	wave 2	232	102.2185	
Phosphors(P) mg/dL	wave 1	21	7.7667	0.697
	wave 2	84	5.8019	
Magnesium (Mg) mg/dL	wave 1	78	2.0059	0.129
	wave 2	69	2.1425	
Calcium mg/dL	wave 1	153	6.2362	0.720
	wave 2	201	5.8431	

## 4 Discussion

COVID-19 is an ongoing pandemic that continues to spread. According to WHO reports, more than 583 million cases have been confirmed globally while 6.44 million people have died as of 2 August 2022 (17). The WHO 2024 updates on COVID-19 have highlighted evolving trends and shifts in case numbers, as well as the development of variants globally. In early 2024, cases increased by 4% in January, showing sustained transmission in some areas despite a decline in deaths (7). After the initial spread of COVID-19 in China, it is evident that, together with age and other risk factors such as comorbidities, alterations in different biochemical biomarkers can be useful for assessing disease severity and the risk of evolution toward critical stages (17).

On 11 March 2022, the first case of COVID-19 was confirmed in Ethiopia. Several studies have shown that age, comorbidities, and abnormalities in various clinical biomarkers are essential for understanding disease severity (18, 19). SARS-CoV-2 infects people of all age groups; however, individuals aged >60 years, along with those with comorbidities such as diabetes, chronic respiratory disease, and cardiovascular diseases, are at a higher risk of developing the infection (19). Biochemical monitoring of patients with COVID-19 is critical for assessing disease severity and progression, as well as for monitoring therapeutic intervention (20).

An overview of the changes in the most common biochemical parameters observed in patients with COVID-19 across waves and multicenter studies is still lacking. Therefore, the present study presents the changes in biochemical profiles, medical histories, and symptoms of patients with COVID-19. In this report, a total of 240 and 298 patients with COVID-19 were studied during the first and second waves in Ethiopia, respectively.

This study found an association between the mean alkaline phosphatase levels of patients across the two waves of COVID-19 and the mean difference was -45.4. It shows that average alkaline phosphatase levels of patients decreased in the second wave. The other biochemical parameter of patients with COVID-19 where themean value significantly differed across the first two waves was the level of sodium. The mean significant difference in the sodium level was −1.883, indicating that the mean sodium level of patients with COVID had decreased in the second wave compared with that in the first wave. This might be due to the changing nature of the virus, population health, and healthcare factors. Several common biochemical parameters have been implicated in the progression of COVID-19, providing important prognostic information (20).

However, the other biochemical parameters of the patients did not significantly differ between the first two COVID waves. Thus, the mean biochemical profiles of patients in the first wave were similar to those of the patients in the second wave.

Chi-square analysis demonstrated that COVID-19 symptoms differed significantly across the waves of the pandemic in Ethiopia. This finding was consistent with that of Suleyman et al. (21).

## 4 Conclusion

This study found that >90% of patients in both the first and second waves of COVID-19 in Ethiopia were not comorbid with chronic lung disease. The mean alkaline phosphatase and sodium levels of the patients significantly differed across the first two waves of the pandemic. Headache, myalgia, chest pain, dry cough, fatigue, fever, joint pain, enlarged glands, rash, shortness of breath, dark or bloody urine, yellow skin/eyes, stiff neck, difficulty in swallowing, wheezing, diarrhea, and chills or rigors were the most common symptoms of COVID-19 in Ethiopia during the first and second waves of the pandemic.

## Data Availability

The raw data supporting the conclusions of this article will be made available by the authors, without undue reservation.

## References

[1] Organization WH. Coronavirus disease (COVID-2019) weekly epidemiological update and weekly operational update. (2020) 2020:.

[2] Fisher D Heymann D. Q&A: The novel coronavirus outbreak causing COVID-19. BMC Med. (2020) 18:1–3. doi: 10.1186/s12916-020-01533-w 32106852 PMC7047369

[3] Buliva E Elhakim M Minh T Nguyen N Elkholy A Mala P. Emerging and reemerging diseases in the World Health Organization (WHO) Eastern Mediterranean Region—progress, challenges, and WHO initiatives. Front Public Health. (2017) 5:276. doi: 10.3389/fpubh.2017.00276 29098145 PMC5653925

[4] Zhong B-L Luo W Li H-M Zhang Q-Q Liu X-G Li W-T. Knowledge, attitudes, and practices towards COVID-19 among Chinese residents during the rapid rise period of the COVID-19 outbreak: a quick online cross-sectional survey. Int J Biol Sci. (2020) 16:1745. doi: 10.7150/ijbs.45221 32226294 PMC7098034

[5] Organization WH. Rational use of personal protective equipment for coronavirus disease (COVID-19): interim guidance, 27 February 2020. World Health Organization (2020).

[6] Organization WH. WHO director-general’s opening remarks at the media briefing on covid-19-11 march 2020, Vol. 2020. (2020).

[7] WHO: World Health Organization. COVID-19 Dashboard. Geneva: WHO (2022).

[8] Kumar R Singh V Mohanty A Bahurupi Y Gupta PK. Corona health-care warriors in India: knowledge, attitude, and practices during COVID-19 outbreak. (2021) 10:. doi: 10.4103/jehp.jehp_524_20 PMC8057180 34084791

[9] Organization WH. Coronavirus disease 2019 (COVID-19): situation report. (2020) 70:.

[10] Surveillances V. The epidemiological characteristics of an outbreak of 2019 novel coronavirus diseases (COVID-19)—China, 2020. China CDC Weekly. (2020) 2:113–22. PMC8392929 34594836

[11] Yuan X Huang W Ye B Chen C Huang R Wu F. Changes of hematological and immunological parameters in COVID-19 patients. (2020) 112(4):553–9. doi: 10.1007/s12185-020-02930-w PMC7354745 32656638

[12] Grasselli G Greco M Zanella A Albano G Antonelli M Bellani G. Risk factors associated with mortality among patients with COVID-19 in intensive care units in Lombardy, Italy. (2020) 180(10):1345–55. 10.1001/jamainternmed.2020.3539 PMC7364371 32667669

[13] Kunno J Supawattanabodee B Sumanasrethakul C Wiriyasivaj B Kuratong S Kaewchandee C. Comparison of different waves during the COVID-19 pandemic: retrospective descriptive study in Thailand. (2021) 2021:. doi: 10.1155/2021/5807056 PMC8519693 34659835

[14] Wang C Deng R Gou L Fu Z Zhang X Shao F. Preliminary study to identify severe from moderate cases of COVID-19 using combined hematology parameters. (2020) 8(9):. doi: 10.21037/atm-20-3391 PMC7290538 32566620

[15] Moradi EV Teimouri A Rezaee R Morovatdar N Foroughian M Layegh P. Increased age, neutrophil-to-lymphocyte ratio (NLR) and white blood cells count are associated with higher COVID-19 mortality. (2021) 40:11–4. doi: 10.1016/j.ajem.2020.12.003 PMC7717776 33333477

[16] Organization WH. Living guidance for clinical management of COVID-19: living guidance, 23 November 2021. World Health Organization (2021).

[17] Organization WH. COVID-19 weekly epidemiological update, edition 99. (2022).

[18] Sarhan AR Hussein TA Flaih MH Hussein KR. A biochemical analysis of patients with COVID-19 infection. (2021) 2021:. doi: 10.1155/2021/1383830 PMC8542065 34703628

[19] Nayak AH Kapote DS Fonseca M Chavan N Mayekar R Sarmalkar M. Impact of the coronavirus infection in pregnancy: a preliminary study of 141 patients. (2020) 70(4):256–61. doi: 10.1007/s13224-020-01335-3 PMC7340730 32760169

[20] Hui DS Azhar EI Madani TA Ntoumi F Kock R Dar O. The continuing 2019-nCoV epidemic threat of novel coronaviruses to global health—The latest 2019 novel coronavirus outbreak in Wuhan, China. (2020) 91:264–6. doi: 10.1016/j.ijid.2020.01.009 PMC7128332 31953166

[21] Suleyman G Fadel RA Malette KM Hammond C Abdulla H Entz A. Clinical characteristics and morbidity associated with coronavirus disease 2019 in a series of patients in metropolitan Detroit. (2020) 3(6):e2012270–e2012270. doi: 10.1001/jamanetworkopen.2020.12270 PMC7298606 32543702

